# The cultural evolution of distortion in music (and other norms of mixed appeal)

**DOI:** 10.1098/rstb.2024.0014

**Published:** 2025-04-03

**Authors:** Gregory A. Bryant, Paul E. Smaldino

**Affiliations:** ^1^ Department of Communication, University of California, Los Angeles, CA 90095-1563, USA; ^2^ UCLA Center for Behavior, Evolution, and Culture, Los Angeles, CA, USA; ^3^ Cognitive and Information Sciences, University of California, Merced, CA, USA; ^4^ Santa Fe Institute, Santa Fe, NM, USA

**Keywords:** music, distortion, cultural evolution, nonlinear phenomena, rock, noise

## Abstract

Music traditions worldwide are subject to remarkable diversity but the origins of this variation are not well understood. Musical behaviour is the product of a multicomponent collection of abilities, some possibly evolved for music but most derived from traits serving nonmusical functions. Cultural evolution has stitched together these systems, generating variable normative practices across cultures and musical genres. Here, we describe the cultural evolution of musical distortion, a noisy manipulation of instrumental and vocal timbre that emulates nonlinear phenomena (NLP) present in the vocal signals of many animals. We suggest that listeners’ sensitivity to NLP has facilitated technological developments for altering musical instruments and singing with distortion, which continues to evolve culturally via the need for groups to both coordinate internally and differentiate themselves from other groups. To support this idea, we present an agent-based model of norm evolution illustrating possible dynamics of continuous traits such as timbral distortion in music, dependent on (i) a functional optimum, (ii) intra-group cohesion and inter-group differentiation and (iii) groupishness for assortment and social learning. This account illustrates how cultural transmission dynamics can lead to diversity in musical sounds and genres, and also provides a more general explanation for the emergence of subgroup-differentiating norms.

This article is part of the theme issue ‘Nonlinear phenomena in vertebrate vocalizations: mechanisms and communicative functions’.

## 1. Introduction

Questions regarding the origins of music have puzzled scholars for centuries. A quick survey of the world’s music reveals a vast array of instrumental and vocal sounds structured by tradition and convention, manifesting in innovative ways across countless social contexts. The relatively recent introduction of electronics in the production and recording of music, along with dramatic innovations in its distribution, has increased this diversity tremendously. One common limitation of evolutionary theories of music is their lack of explanation regarding the incredible sonic variation in what we observe in contemporary cultures—an issue because this variation is arguably one of the most interesting and defining features of musical behaviour. Evolutionary accounts propose adaptive solutions for functions such as coalition signalling [1–4], parent–offspring communication [5], social bonding [6], predator deterrence [7,8], sexual signalling [9,10] and more, but these factors cannot explain in any detail how and why you get a Bach chorale, a South African drum circle, or a dissonant noise drone in the times and places they appear. An important part of the puzzle is missing.

We define music as a varied category of sound-based, intentionally produced performative activity, typically embedded within a cultural milieu. In many languages, there is no word that maps directly to the English word ‘music’. Instead, the closest concept often refers to a cultural activity that includes phenomena some might describe separately as music. This fact reveals not only that the practice varies substantially across cultures and time, but that its conception does as well. In the West, we have decomposed music to its smallest constituents—for scholarly reasons of analysis and teaching, and production reasons related to music creation, performance and perception. Through technology, we have developed techniques for fine control over every parameter we can imagine.

Human musicality, defined broadly as the collection of traits that contribute to the production and perception of music, integrates many different cognitive, perceptual and behavioural systems [1,11,12]. Music itself can be construed as a family of socially learned behaviours, subject to normative constraints, that incorporates the generation of intentional sounds through instruments and singing. Cumulative cultural evolution guides the ongoing manifestation of music and should be seen as a complementary level of evolutionary analysis for explaining the implementation and variation of musical behaviour through history and across cultures. Many specialized abilities important for music production and perception evolved for nonmusical reasons, most being older than humankind and even hominoids. For instance, mechanisms supporting auditory scene analysis allowing for sound stream segregation and localization are highly relevant for how we perceive music but exist in highly conserved forms across many terrestrial animals [13,14]. Similarly, the form–function relationships between vocal signalling and affective intent occur across mammals [15,16] and are reflected in clear ways across the world’s music, including emotional vocal performances of singers and instrumental emulations of emotional voices [1,17–19]. But while animals such as songbirds and whales express cultural variation in particular melodic features of their signalling [20], the sound of human music varies to an extreme degree: from soft and slow to loud and fast, from highly rhythmic melodies to nonrhythmic drones, from dulcet to distorted. What is the source of this variation?

We propose that much of this sonic and stylistic variation can be explained by appealing to the tension between the needs of a collective to organize and coordinate around culturally specific activities, and the needs of its members to differentiate themselves from other groups. The former dynamic is likely to be shaped by the utility of certain sounds in evoking reactions that are beneficial to particular activities, while the latter dynamic should be particularly important in large, diverse populations (e.g. WEIRD) and may help explain the continuous adaptive radiation of diverse musical subgenres. In the remainder of this article, we flesh out our argument and present a simple formal model of cultural transmission dynamics that can be applied, in theory, to any musical feature that is not strictly necessary for music production and can have mixed aesthetic appeal. While we focus on music (and specifically musical distortion), our model is actually much more general and applies to the cultural evolution of a wide range of normative behaviours, both musical and nonmusical. In the long run, the cultural coevolution of group identities, technologies and normative signalling and coalition-building practices can produce group practices whose styles serve at once to unite group members and deter or baffle outsiders.

**Figure 1.**
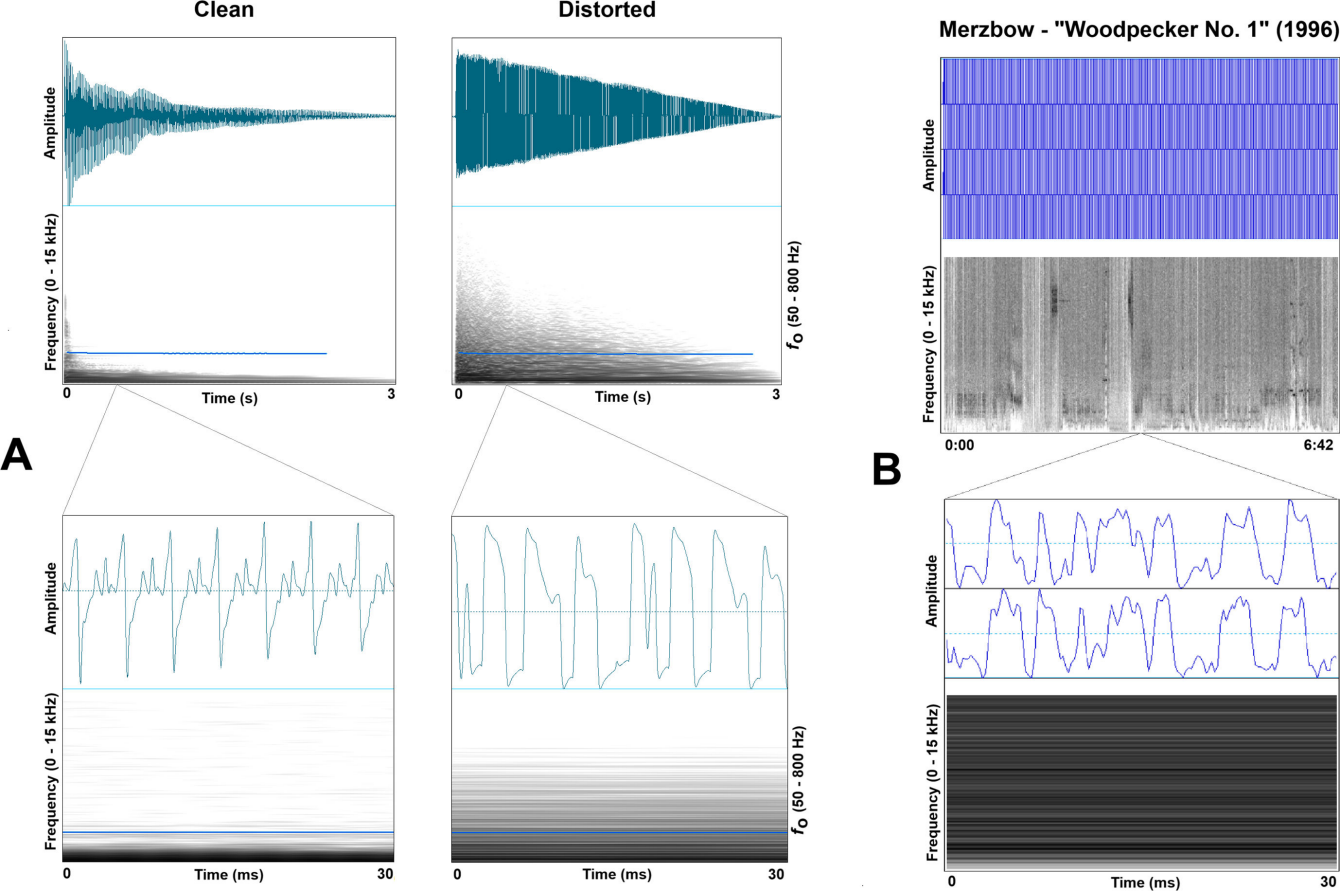
Musical distortion examples. A) Upper panel: Mono waveform and narrowband fast Fourier transform (FFT) spectrogram (50 ms Gaussian analysis window, 44.1 kHz sampling rate and 0−15 kHz frequency range) of a single, plucked electric guitar string (E: 82.4 Hz) clean and distorted (King of Tone pedal by Analogman, Distortion mode). Lower panel: 30 ms segments taken from each at 500 ms. (B) Upper panel: Stereo waveform and narrowband FFT spectrogram (50 ms Gaussian analysis window, 44.1 kHz sampling rate and 0−15 kHz frequency range) of ‘Woodpecker No. 1’ by Merzbow recorded in 1996 illustrating a 6 min atonal noise composition lacking discernible rhythm and melody. Lower panel: 30 ms segment taken at approximate halfway mark (3:20).

Here, our model is intended to describe any facet of music that occurs variably across traditions. One example is musical distortion (including fuzz, roughness and other spectral noise) that appears widely in music from industrialized populations, most prolifically in rock and related genres, but which has also manifested sporadically and variably in music across cultures and time. Distortion can be technically defined as the progressive transition of a sound wave (for an electric guitar, a triangle wave) perceived as relatively ‘clean’, into a square wave as a function of amplitude gain ([21]; see figure 1A).

This nonlinear process [22] typically results in degradations of periodicity with preserved pitch, combination tones, and at extreme levels can result in atonal broadband noise (figure 1B). Analogue circuitry and digital algorithms can affect a transformation in infinite ways through clipping, compression and filtering of the sound wave, which can introduce upper harmonics and allow sound designers to create timbral palettes that afford a vast array of musical characteristics. We suggest that this class of distortion phenomena in music has been culturally shaped by a perceptual attractor to so-called nonlinear phenomena (NLP; described in detail below). Musical distortion triggers mechanisms evolved for detecting intense affect in voices of social agents signalled through NLP. In turn, distortion features in music have been culturally selected and significantly refined to be generated in contexts that do not necessarily reflect any reliable physiological correlate; that is, they are not typically indexing real embodied arousal but instead are intentional and often honest signals of aesthetic preference. The resulting distorted sound characteristics in music have been shaped by cultural evolutionary forces as a means of not only enhancing sounds of instruments and voices but also differentiating genre subcultures and the agents who participate in them. In the following sections, we will describe (i) the relevant principles of cultural evolution, (ii) a cultural evolutionary approach to understanding the music faculty, (iii) a background of NLP in vocalizations and distortion in music and (iv) an agent-based model of cultural trait evolution with an eye toward the proliferation of musical distortion.

## 2. Cultural evolution

Cultural evolution is a rich, formalized theoretical framework for understanding cultural stability and change [23–25]. Extending the principles of Darwinian natural selection to include the social learning and cultural inheritance of behaviours, ideas and technologies, the field has developed over the past four decades empirically supported formal theories of information transmission, cooperation, norms, identity signals, intergroup competition and more. Particularly important in understanding human culture is our capacity for *cumulative* cultural evolution—the population-level ability to refine, extend and recombine technological and cultural innovations over generational time [26–28]. Moreover, many of these cultural innovations are collective behaviours that involve the normative coordination of numerous individuals. Music is such an innovation. As such, the fitness advantages stem not only from engaging adaptively with one’s environment but also from the ability to effectively cooperate and thereby produce the sorts of synergistic benefits that only groups can produce [26,29].

Cultural evolution is possible because human psychology permits traits to be transmitted by a second channel alongside genetic transmission: social learning. Indeed, seminal work in the field set out to understand what sort of learning psychology could be shaped by natural selection to permit adaptive cultural transmission [23], and a wide range of social learning strategies has been identified that increase the adaptive value of social information [30]. Preferentially, learning from successful or high-prestige individuals is often an effective way to maintain useful skills in a population, though diversity can limit the adaptive value of that information if it originates from someone in dramatically different circumstances than one’s own. In this case, signals of group identity can direct one’s attention toward information sources likely to be most useful, particularly when identity information is coupled with information about success or prestige [31]. Signals indicating group identity, including indicators of musical subcultures in contemporary industrialized societies, are key drivers of cultural evolution, facilitating within-group coordination and bolstering between-group differentiation [32–34]. Clear group boundaries are well-known drivers of cooperation and effective management of collective resources [35].

The cultural evolution of musical distortion taps into at least three factors contributing to the social transmission of behaviour. The first is the need to capture the attention and aesthetic appeal of an evolved human psychology. Music engages with numerous physiological and psychological processes and would not persist if it did not facilitate behaviours that were adaptive more often than not. Local social ecologies will also play a role in shaping the optimal aspects of music. Musical sound features have a form–function relationship with affective reactions and likely subsequent behavioral responses [1]. For example, fast rhythms coupled with musical distortion are particularly effective at inducing intense emotional responses and physical movement (e.g. slam dancing), whereas lullabies, for instance, should not have these properties given the communicative functions of singing to children [36]. Aspects of music that provide a better fit to needs of human psychology and the local ecology will therefore lead to greater success and more widespread adoption. The second is the need to coordinate with others. Music provides a benefit for interactivity, coalition-building and coalition-signalling only if members of a community agree on the norms of how music is produced and engaged with. Aspects of music that conform to the norms of the community will therefore lead to greater success and more widespread adoption. The third is the need to strengthen group boundaries by differentiating the norms of one’s group from those of other groups. Music serves a signalling and coordinating function by helping to demarcate group commitments and allegiances. In this context, distortion may particularly benefit from its abrasive sonic characteristics—individuals who are not enculturated in a group’s music containing distortion may have a difficult time benefitting from participation.

## 3. Origins of the music faculty

Music worldwide has two primary features almost without exception: rhythm and melody. Rhythm comprises auditory events that provide a beat—often isochronic in repeated and stable temporal intervals—and typically with accented elements (i.e. strong and weak beats). Melody refers most generally to modulations of pitch within a hierarchy of tones. Together, these two features characterize structured musical behaviour, but many other components of music are specific to a given place and time, including instruments, production methods, social functions and so on. These variably appearing features of musical activity provide a window into possible biological and cultural mechanisms underlying music more generally [36,37].

As mentioned above, specific musical features can usually be attributed to cognitive, perceptual or behavioural adaptations solving recurrent adaptive problems outside of the domain of music. For example, language and speech systems likely underlie many aspects of music traditions, such as hierarchical structure in rhythms and melody, transposability and generativity [11,38], as well as obvious roles in lyrical singing and written music notation. Abundant evidence suggests voice perception mechanisms give rise to musical scales [39], major and minor modes [40], consonance and dissonance [41,42] and musical intervals [43–45]. Biomechanical systems afford the construction and use of musical instruments [46], and memory systems allow for the learning of musical compositions for repeated performance and cultural transmission. These are just a sample of plausible connections between multicomponent mechanisms and the widespread features of music around the world.

But there are also theoretical reasons to assume the existence of music-specific mechanisms. For example, action-perception coupling allowing for social entrainment (i.e. shared spatiotemporal, motor-based coordination between independent agents) is intimately tailored to afford coordinated signalling by groups, a unique species-specific behaviour [1–4,7,47]. The production and perception of infant-directed melodies also can be considered as a musically based signalling system solving problems of caregiver–infant communication [3,5,48].

Regardless of the origins of any mechanism within the music faculty, all musical features are subject to the forces of cultural evolution. Moreover, the iterative nature of cumulative culture can generate highly exaggerated forms, analogous to ritualization that characterizes the co-evolutionary processes shaping the design of animal signalling systems [49]. Our focal example in this paper is the cultural evolution of musical distortion. While distortion can currently be produced in myriad ways—with tools including amplifiers, effects pedals, synthesizers, megaphones and computer software—it is likely derived from NLP in voices, and through cumulative culture has evolved into an amazing diversity of chaotic sonic phenomena.

## 4. Nonlinear phenomena in vocal communication

NLP occur widely in vocal behaviour across species. There are a variety of acoustic phenomena that theorists have described as nonlinear including subharmonics, harmonic sidebands, biphonation and rapid pitch shifts [50]. All of these complex features appear in human voices and can be musically relevant. But here we focus on the class of phenomena sometimes called ‘deterministic chaos’ in the vocalization literature, where the term is used more narrowly than in the physics and complex systems literatures (e.g. [51]). Deterministic chaos in this context can be defined as broadband-like noise due to chaotic vibration regimes in the vocal apparatus generating aperiodic waves. It is worth noting that *all* aspects of vocal production are subject to nonlinear dynamics, including the transition from a resting state to a sound-producing mode. But deviations from the steady dynamics of ordinary control often result in distinct modes of production with perceptual, communicative and adaptive consequences. It is also important to note that musical distortion is generally *not* produced through chaotic processes (outside of natural vocal production), but instead the relevant distortion sounds in music are emulated through various technologies (e.g. wave shaping, synthesis, amplification, etc.). It is these phenomena that are of interest here, and particularly their ability to serve as targets for cultural selection.

Subjectively, deterministic chaos in vocalizations has a distorted, harsh sound resembling broadband noise and appears on a spectrogram as widely distributed energy across the frequency spectrum. Chaotic vibration regimes in vocal systems can arise due to excessive energy being forced through the glottis and vocal tract. In behavioural contexts of high arousal, animals (including humans) will produce vocalizations of high energy, resulting in distorted growls, yells or roars. These acoustic features allow receivers to extract important behavioural and ecological information and have, over time, led to adaptive perceptual responses. If an animal is experiencing high arousal, it is crucial for others to recognize it and act accordingly, such as preparing for attack, responding to an alarm or expressing submission.

Some nonlinearities have likely been ritualized into extremely salient properties of signals. NLP have features that are particularly effective at getting others’ attention and are difficult to habituate to (e.g. [52]). These properties allow NLP to have important communicative effects, making them a target for selection in communication systems [50]. Examples abound but consider the role of NLP in human baby cries. The high arousal underlying infants’ crying manifests itself as high-energy output in crying vocalizations [53]. Babies cry for a variety of reasons, most related to getting caregivers’ attention and influencing them to provide basic needs. Crying is shaped by selection for perceptual averseness, which motivates caregivers to take action that makes it stop, with different acoustic properties indicating the severity of need [54]. The chaotic elements in crying honestly reflect high negative arousal, and the sound itself is particularly unpleasant to hear, providing the basis for selection to act on the vocal features to further enhance their effect. It is often in receivers’ best interest to be manipulated by senders (particularly when they are close kin; [55]), and physical forms are selected because of their reliable communicative functions.

Vocal communication plays a large role in most musical traditions. Beyond singing, many instruments emulate vocal sounds to some extent, having similar spectral outputs and incorporating subtle effects that capture aspects of vocal behaviour. The importance of vocal signalling for music is also revealed in the role of vocal learning for the evolution of rhythmic entrainment [56]. Being able to control the voice both individually and in coordinated ways with others could be the most important factor in how musical evolution got off the ground, affording group-level activity. This ability plausibly laid the groundwork for the forces of cultural evolution, and led to remarkable variation in coordinated sound-based traditions.

## 5. Nonlinear phenomena in music

The subjective awareness of various types of noise and dissonance in music has long fascinated perceptual psychologists, musicologists, computer scientists and behavioural biologists. For example, Helmholtz [57] performed early psychoacoustic studies on people’s perception of roughness in consonant and dissonant music intervals, with prescient remarks about the physiological factors that contribute to roughness judgements and how these features could be used for musical expression and subject to cultural change. Given the prevalence and salience of NLP in human vocalizations, we should expect them to be present in musical cultures. Indeed, we can find a variety of NLP across singing traditions. For instance, the ‘jing’ role in Chinese opera (i.e. a forceful male character) is characterized by a growl-like quality. Tsai *et al.* [58] described the jing style and explicitly drew connections to evolutionary approaches to animal signalling, noting the high arousal and negative affect linked to noisy growling styles. Sakakibara *et al.* [59] used videofluoroscopy and high-speed imaging, along with acoustic analysis and model simulation to describe various traditions worldwide that incorporate growl components in their singing. These researchers examined *umngqokolo*, a Xhosa vocal tradition in South Africa, *kakegoe* in Japanese theatre, American jazz singing (e.g. Louis Armstrong) and drone throat singing of Tuva (also see [60]). We also find various NLP in contemporary vocal music [61], and many researchers have documented the ‘rock growl’ (e.g. [62]). Herbst *et al.* [63] described different terms used by researchers for vocal production modes associated with vibrating ventricular folds (sometimes called ‘false’ vocal folds), and vibration regimes of other supraglottal structures, including growl, dist, throat-singing and distortion.

NLP also appear across many musical traditions in non-vocal instruments, including traditions without electronics. For example, chaotic sound features are present in the jinghu, a traditional Chinese bowed instrument that can be manipulated to produce what players sometimes refer to as a ‘tiger tone’ containing broadband noise measurable as low harmonics-to-noise ratio [58]. African lamellaphones (e.g. mbira) often include metal attachments such as rings and bottle caps that add highly salient noisy, timbral components [64]. Font-Navarrete [65] noted similar approaches with attached objects on bàtá drums, as well as the practice of wearing noisy rattles on the wrist in bugarabu and tantango drum traditions. In Indonesian Gamelan that includes many percussion instruments such as xylophones and gongs, instruments are manufactured in pairs and tuned to slightly different frequencies so when struck together produce what is called ‘ombuk’, referring to a wave containing an acoustic beating resembling dissonance intervals in Western terminology [66]. Some instruments (e.g. gong ageng) can produce ombuk on their own without accompaniment. The resulting sonic quality is unique to Balinese gamelan music. Examples in Western music include the nonlinear modes of the clarinet and saxophone that can introduce quasi-periodic and even chaotic states as a function of amplitude [67,68], period doubling in trombone, crumhorn and bassoon [69] and subharmonics in violin [70].

These examples are not exhaustive of course but illustrate the widespread use of NLP in instrumental sounds that are used not only as a means of musical expression but as a signature sound unique to the musical tradition in which they culturally evolve. These acoustic phenomena do not appear out of nowhere but instead likely often manifest as by-products of an instrument’s design, and then become developed and enhanced because of their contribution to the played sound. A variety of stories exist regarding the introduction of distortion in rock, swing and blues over the last century, including tales of damaged speaker cones occurring during transport, and overblown low-fidelity amplifiers that were regularly turned up too loud [71]. In these cases, the accidents resulted in guitar sounds with a gritty and fuzzy appeal. Players soon began purposefully altering their amplifiers and speakers, experimenting with the effects. The added textural complexity became a signature sound for some artists and allowed affected instruments to become more salient (i.e. ‘pop out’ of the mix) and add a slightly more intense element to performances. Both of these effects correspond to the signalling functions of NLP in vocalizations. This is a crucial component of how musical sounds emerge and evolve and requires an explanation that incorporates the dynamics between individuals interacting in musical contexts, including performers and listeners.

As in traditional instruments and singing styles, noisy sound phenomena in rock and blues usually first occurred by chance but were subsequently highlighted in various strategic ways. The unique story of rock music, however, is that the effects evolved in what could be described as runaway selection [72]. It was not long before amplifiers were purposefully designed to have overdrive, allowing for a distorted sound without damaging the equipment or turning up the gain excessively. Arguably, the biggest development for creating distortion was the invention of the fuzz pedal in the early 1960s, which took a clean signal directly from the instrument and distorted it with added inharmonic overtones. The proliferation of pedals happened quickly, with the first ‘distortion’ pedal appearing in the late 1970s, followed quickly by the emergence and widespread adoption of distortion, fuzz and overdrive pedals, rackmount units and post-production digital tools that could be applied to any sound. Overall, these tools usually work by generating an amplitude gain accompanied by a low-pass filter and pushing the signal toward a saturating nonlinearity such that increases in input gain do not map in a linear way to the output amplitude. The resulting transformation is filtered again, becoming a multi-band-passed nonlinearity. The sound of this process applied to a tone is noisy and distorted, with newly introduced inharmonic spectra. This kind of signal transformation can be used in endless, fine-grained ways to colour any input one might choose to manipulate. Soon, music artists would be adding distortion effects to every sound imaginable.

A distinct power of distortion is the huge versatility in how the effect can be implemented, providing fodder for artists to carve out unique sounds. This new means for self-distinction was the fuel for a cultural revolution of sound palettes [73], and a source of musical cultural evolution. A description of the last 70 years of distortion effects in rock music is beyond the scope of this article, but one important aspect should be noted. The use of distortion in music has ratcheted up continuously, with relatively subtle overdrive and fuzz effects appearing (accidentally) before the 1950s, purposefully and more noticeably in the 1960s and 1970s, and then taking on a life of their own in the 1980s and beyond. Many strands of noisy elements infiltrated modern music, even in the early twentieth century—the advent of avant-garde noise composition (e.g. musique concrète) predates modern rock music [74]. While historically relegated to the extremes of cultural awareness, recent artists have brought noise slightly more into the mainstream [75]. Noise music is at times without rhythm or melody, manifesting as sounds many people today would not classify as music at all, and certainly not resembling anything most people throughout history would ever consider to be musical (e.g. Merzbow, Sunn O))), Wolf Eyes, among many other artists; see figure 1B). An explanation of musical behaviour needs to account for this phenomenon—a model is needed that can demonstrate how some music features will likely spread to all populations in short time frames, and others might only spread very selectively in specific niches.

NLP have predictable effects on people’s emotional interpretations of music. An analysis of movie scores found several NLP were used most frequently in the horror genre and did so in specific ways to elicit a scary effect (e.g. screechy sound in the *Psycho* theme; [76]). In follow-up work, musical distortion and rapid frequency shifts were manipulated in musical compositions, and the pieces were played for listeners who rated them for affective content [77]. The music was composed to be emotionally neutral, including not being in obvious minor or major modes, in medium tempi and in average amplitude ranges. The excerpts were 10 s in duration, and NLP were added at the 5 s mark, including musical distortion and abrupt frequency shifts in the melodies. In a within-subjects design, listeners heard versions either with or without the added NLP and were then asked to judge the arousal and valence in the tracks. A second study had different listeners rate the same audio tracks, but they were paired with benign videos that portrayed simple scenes with actions occurring at the 5 s mark (e.g. turning a page of a newspaper or getting up from a seat).

As expected, compositions with distortion added were judged as having higher arousal and more negative valence than their unmanipulated counterparts. Rapid upward pitch shifts also caused perceptions of greater arousal, and downward pitch shifts caused more negative judgements of valence. In the second experiment with paired videos, the effect of distortion on arousal disappeared, but valence judgements were relatively more negative. Downward pitch shifts also caused more negative valence judgements, similar to the first audio-only study. These results were interpreted as showing how arousal-linked signals might be discounted in multimodal contexts where visual experience does not suggest an immediate threat. In another study [19], listeners judged vocalized vowels (/a/) presented in different contexts (no context, white noise, clean guitar and distorted guitar). The vowel sounds depicted three levels of anger (low, medium and high), with nonlinear features increasing as the anger portrayals intensified. Vocalized anger was not perceived differently across the contexts. Instead, the global occurrence of distortion in voices and instruments was related to percepts of higher arousal and negative valence. These studies and others (e.g. [17,78,79]) suggest that NLP in instruments are processed in a multisensory context so that their interpretation is coloured by the attentional and behavioural demands of the situation. This accords with a recent study of guitarists by Herbst [80], indicating that preferences for more or less distortion were largely aligned with the norms of the musical genres in which the guitarists typically played. Anecdotally, distortion cannot really evoke only negatively valenced emotional responses, otherwise it would be difficult to explain the popularity of distortion-heavy music. Instead, such preferences can be learned as part of enculturation, much as people learn to enjoy the taste of spicy foods despite the reliable activation of pain receptors by capsaicin [81].

The strength of the attractor on which a given musical feature is based should be a major determinant of how widespread that feature will proliferate in a population of music variants. Distortion, along with other musical features, serves important social functions. It increases arousal and as such can foment entrained group cohesion coupled with rhythm and melody. The commonly experienced negative valence of this arousal can be viewed as a positive feature in many contexts. For example, if a group or subculture wants to differentiate themselves from other groups (including the dominant mainstream culture), they may do so through music that requires some degree of enculturated desensitization and which is therefore often unpleasant to those outside the culture. In Western music of the last few decades, punk, metal and rave scenes all fit this description.

Musical features are malleable, cultural contexts for musical experiences vary and groups need to distinguish themselves from others. These factors all contribute to the extent to which we should find variation or universality in the characteristics of music across genres and cultures. In the following section, we present an agent-based model that formalizes and fleshes out this argument and allows us to begin to analyse the cultural transmission dynamics underlying the proliferation of behavioural traits, including music features that can spread through populations.

## 6. A cultural evolutionary model of distortion in music

Our model is effectively one of norm evolution, using norms with continuous values that can represent any continuous behavioural trait, such as the extent of timbral distortion in music. The model is loosely based on a recent model of continuous norm evolution by Yan *et al.* [82] that considered traits in which the drivers of pay-off were similarity to peers’ trait values and closeness to some objectively optimal value. To this, we add additional incentives to differentiate one’s trait value with values associated with an outgroup, as well as coalitional assortment by which we vary the extent to which agents preferentially interact with and imitate ingroup individuals. The importance of intra-group cohesion is driven by the relative importance of coordinating with interaction partners coupled with the propensity for those partners to be drawn from one’s ingroup. By varying the relative strength of these influences on trait values, we can capture the relative importance of (i) a functional optimum, (ii) intra-group cohesion, (iii) inter-group differentiation and (iv) groupishness for assortment and social learning.

### (a) Model design

We consider a population of *N* agents, each belonging to one of *M* groups, which for the present paper we limit to two (*M* = 2). Each agent *i* is characterized by a real-valued trait, *x_i_*. Agents increase their pay-off when they interact with others with similar values, representing intra-group cohesion. The trait’s value may also be optimized for some functional purpose when it is closest to the objective value, *θ*. Yan *et al.* [82] show that when these are the only two operative forces, the population will always converge to a single normative trait value if *θ* is the same for all agents. To these forces we also add the utility of outgroup dissimilarity, which increases with the distance between *x_i_* and the mean trait value of the outgroup. We will show how these forces can drive the cultural evolution of group-distinct trait values.

We initialized the model with two groups exhibiting very minor differences. Our aim is to determine when group differences emerge. In future work, we want to allow group identities to emerge endogenously; for now, they are assigned at initialization. All agents had their initial trait values drawn from a normal distribution with a mean of *μ*_*g*_ and a standard deviation of 1, where *g* corresponds to the group. Our simulations were run with *μ*_1_ = 0 and *μ*_2_ = 1, allowing for small but noticeable initial differences between the groups while maintaining both variation within groups and similarity between them. A proportion *p* of agents was in group 1, and the remainder in group 2. For our simulations, we assumed equal group sizes, *p* = 0.5.

The model dynamics proceed in discrete time steps, each of which involves two stages: interaction and imitation. During the interaction stage, each agent *i* first selects an interaction partner *j*. With probability *r*, representing the strength of coalitional assortment, this partner is chosen from the set of agents in the focal agent’s ingroup. Otherwise, the partner is chosen from among the entire population at random, so that *r* = 0 represents a well-mixed population. The pay-off to agent *i*, *V_i_*, is calculated as follows:


(6.1) $$V_{i}=e^{-\alpha {\left(x_{i}-\;\theta \right)}^{2}}e^{-\beta {\left(x_{i}-\;x_{j}\right)}^{2}}+\left(1-e^{-\gamma {\left(x_{i}-{\underline{x}}_{out}\right)}^{2}}\right),$$

where *θ* is the objective optimal trait value, ${\underline{x}}_{out}$ is the mean trait value of agent *i*’s outgroup and *α*, *β* and *γ* are the relative weights of the objective value, partner similarity and outgroup dissimilarity, respectively.

During the imitation stage, each agent chooses a target to imitate. An agent is chosen as a target with a probability proportional to its pay-off from the interaction stage. With a probability *ρ*, the focal agent restricts the set of possible targets to members of its own group, representing the strength of coalitional copying.

Our analyses focused on the differences between the median trait values of the two groups. Values close to zero indicate convergence to a universal standard value, while deviations from zero indicate the emergence of group-specific norms. For the preliminary analyses presented here, we studied a range of values for the weights of objective value and partner similarity, while keeping the weight of partner dissimilarity fixed at *γ* = 0.05. As Yan *et al.* [82] demonstrated, the population will *always* converge to a single value if *γ* = 0. We do not report a full sweep of the model parameters here; the value of *γ* = 0.05 was selected as a minimal round number that generated stable between-group divergence, but visual inspection indicates that a wide range of values yield similar results. We ran simulations for 100 steps, which we determined by visual inspection was well above the time needed for the model to reach an equilibrium. All results are averaged from 30 runs for each set of parameter values. The model was coded in NetLogo 6.4. Code is available as part of the electronic supplementary material.

### (b) Simulation results

**Figure 2.**
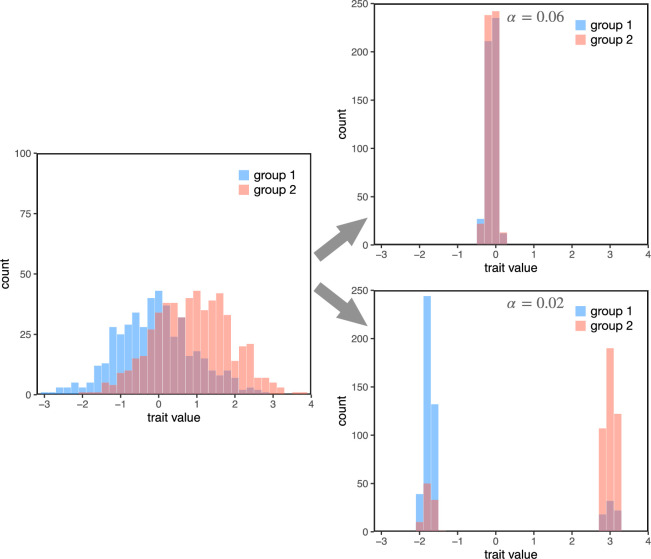
Example model dynamics. At the start, trait values are widely dispersed, with group 2 having a slightly higher mean value (left). When an objective value of zero is more highly weighted (*α* = 0.06), agents’ trait values converge to near zero regardless of group identity (top right). When objective value is not highly weighted (*α* = 0.02), groups diverge widely, with each group’s traits clustered around a new mean (bottom right). Because agents still occasionally interact with and copy from members of other groups, some agents continue to hold trait values more characteristic of their outgroup. For these simulations, *p* = 0.5, *r* = 0.5, *ρ* = 0.75, *β* = 0.5 and *γ* = 0.05.

Figure 2 illustrates how the model dynamics tend to unfold. The left side of the figure shows the initial trait distributions for each group. When the objective value is weighted relatively heavily (*α* = 0.06, top right), the population converges to a single trait value. However, when the objective value of the trait is less important than cultural concerns like intra-group coordination and inter-group differentiation (*α* = 0.02, bottom right), the population splits into two stable trait values, each associated with one of the two groups. Notice that, because assortment is imperfect, some individuals may end up taking on trait values that are more common in the outgroup.

**Figure 3.**
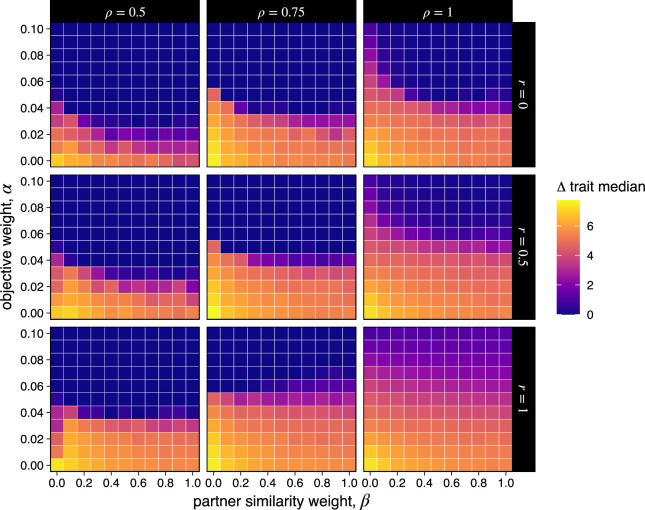
Median trait differences between groups. Average difference between the median trait values of the two groups across 30 runs for each set of parameter values. Differences are sensitive to the objective weight, *α*, the partner similarity weight, *β* and the extents of coalitional assortment (*r*) and copying (*ρ*). For all runs, *p* = 0.5 and *γ* = 0.05.

Figure 3 illustrates how the model parameters affect trait convergence or divergence. In particular, the graphs show how the importance of adopting specific trait values (*α*) and adopting similar trait values as one’s social partners (*β*) affect the average divergence in trait values between the two groups (Δ trait median), for several values of coalitional assortment (*r*) and copying (*ρ*). The most obvious result is that when the absolute value of a trait is very important, we get universal convergence for the trait across groups. This makes sense—when a particular feature needs to be acquired exactly right, there is less room for variation. When this objective value matters less (though it need not be totally inconsequential) and individuals are incentivized to distinguish themselves from outgroup individuals (which we assume for all reported simulation runs, *γ* = 0.05), stable between-group differences can emerge. The relative importance of partner similarity is fairly small; more important is whether those partners are members of the ingroup and whether trait values tend to be copied within groups—both of these factors make between-group divergence more likely. When groups are relatively cohesive, group differences can emerge and persist, with the magnitude of those differences being maximized when both objective value and partner similarity are small in comparison to the importance of outgroup differentiation.

These results help illustrate some important features of the cultural evolution of normative traits such as distortion in musical practices. When cultural differences arise between groups, it indicates either that the purpose of the trait is predominantly to facilitate social coordination or signalling (as in our model), or that each group is using music for a different function, perhaps corresponding to differences in the local ecological pressures to which each group is responding (modelled as a different value of *θ* for each group, which we do not extensively analyse here). When traits serve a coalitional function, between-group differences could arise by drift. If the groups interact, however, within-group coordination is not sufficient to explain variation [82]. The need for coalitional *differentiation* is key. Groups become different because their norms help signal group identity, and this signalling function works best when it reduces ambiguity in determining group membership [83,84]. Returning to distortion, the model dynamics indicate that we can expect groups and subcultures to adopt distinct forms in their music when they need to highlight their differences from other communities.

The model is very simple, involving only two fixed groups and a single continuous trait. It captures neither the continued fragmentation of subgroups nor the increasing differentiation of traits, both of which have been observed in the adoption and evolution of distortion in Western music since the mid twentieth century. However, the model *does* suggest how such a dynamic could occur. As populations grow and groups continue to subdivide due to divergence in interests, goals or circumstances, iterations of the core model dynamic could occur in a fractal nature with each fissioning. The model also does not capture the multidimensional nature of musical traits. Future modelling work can and should tackle this problem, ideally aided by data to help explain why particular patterns of similarities and differences in musical characteristics among historically and culturally connected musical traditions have evolved.

## 7. Discussion

Cumulative cultural evolutionary processes can help explain the incredible diversity of music observed around the world that other evolutionary accounts cannot. Musical innovation can be conceived as the incorporation and successful transmission of phenomena that rely on cognitive, perceptual and behavioural mechanisms (i.e. attractors) that were largely shaped to solve adaptive problems unrelated to music [85,86]. Different musical features are remembered and copied by individuals in a social network and then proliferate at some rate depending on the strength of the attractor they are linked with. In the model described here, we can observe how simple dynamics due to variations in social assortment and copying practices can result in variations in how a trait evolves in a population.

Research examining social functions of music has demonstrated a variety of ways that music operates as a proxy for social assortment in infants and young children [87,88], adolescents (89) and adults [90,91]. There is a clear sensitivity to shared musical culture that drives preferences for friends and allies, and researchers have documented the propensity to copy music listening choices from highly regarded peers and family [92,93]. Moreover, shared tastes in music uniquely correlate with other shared behaviours [94]. Music recommendation algorithms are becoming increasingly sophisticated in this regard, with designers becoming highly sensitive to social network structure when making suggestions for content to users [95]. The model proposed here provides a cultural evolutionary mechanism by which musical features might spread through a socially structured population and suggests a means for shaping the incredible variability we see in musical culture.

The musical feature we focus on here is distortion, which we define broadly as a family of noisy sonic phenomena resulting from transformations of sound waves through heavy amplitude gains and other processing. Technological innovations have introduced distortion into many elements of music, altering the sound features of instrumental and singing performances. A number of related NLP are likely subject to similar perceptual processing and subsequent cultural dynamics, including manipulations of low harmonicity, subharmonics and frequency jumps, all of which can play a role in music. We argue that the attraction to these manipulations is derived from NLP in vocalizations, which have been shaped by selection to elicit attention, resist habituation and signal high arousal and negative valence. Modern distortion is an emulation of NLP, retaining important attributes that share many of the same perceptual effects. One can conceive of the technological developments underlying distortion tools as a kind of culturally mediated psychoacoustic modelling. Distortion often adds compelling texture and nuance to sounds that can be highly provocative to listeners. Importantly, distortion effects can be used in creative ways to get unique sounds that help groups distinguish themselves from those around them. Distortion thus becomes a tool for signalling group identity, subject to the dynamics of cultural transmission. These processes often manifest themselves in ways that highly resemble co-evolutionary ritualization that shapes the structure of many animal signals [49]. Ritualization often results in extreme features, an aspect that is well demonstrated by the cultural evolution of distortion and noise in modern music.

This work adds to a growing literature showing how listeners’ preferences can influence the cultural evolution of specific musical features that can emerge from prior sound-making technologies that do *not* initially have those features. For example, MacCallum *et al.* [96] constructed a music engine that played brief, non-musical sound clips to listeners who then judged them in a forced-choice task. Clips that were preferred were allowed to ‘reproduce’ by recombining with other preferred clips, and others were eliminated. After 1000s of successive generations, distinctly musical attributes emerged, including an isochronic beat. In the domain of rhythm specifically, researchers demonstrated through an iterated learning paradigm (eight generations repeated six times), randomly presented sound sequences evolved into structured rhythmic patterns that became easier to learn over time, isochronic, hierarchically structured and converged on universally observed durational categories [97]. Popescu & Rohrmeier [98] used a similar approach to examine the emergence of melodic structure. These studies uncovered features of rhythmic and melodic phenomena observed cross-culturally [37]. Our model predicts that when human psychology entails a strong, universal preference for (or aversion to) particular sonic phenomena, those phenomena are unlikely to distinguish different cultural and subcultural traditions. Rather, it is the presence or absence of features that capture the attention while producing a *mix* of positive and negative reactions that are most likely to evolve as group-defining cultural markers.

Our approach focused on distortion in music as normative behaviour for intra-group cohesion and inter-group differentiation. In this way, it is related to more general proposals for the emergence of group differentiation and the emergence of markers of group identity [99–102]. But our model is the first to integrate objective value, in-group coordination and out-group differentiation in a way that provides a coherent theoretical explanation for why certain musical (or nonmusical) features are nearly universal, others correspond to local ecologies and others are used to distinguish between co-located cultural communities. Another important driver for inter-group variation, which we have largely ignored here, is socioeconomic status. Style and fashion aesthetics can be driven by elite innovation to distinguish themselves from the general populace or by counter-dominance signalling among communities trying to distinguish themselves from the *status quo*, whose prestige can then be co-opted by the larger population [103]. Inequality in contemporary societies also drives asymmetries in the dissemination of styles and ideas via differential control of information architectures [104], which is likely to further shape the cultural evolution of artistic styles. While the essentials of our model are recapitulated in more complex, hierarchical societies, the contribution of this additional social structure is an important topic for future research.

**Figure 4.**
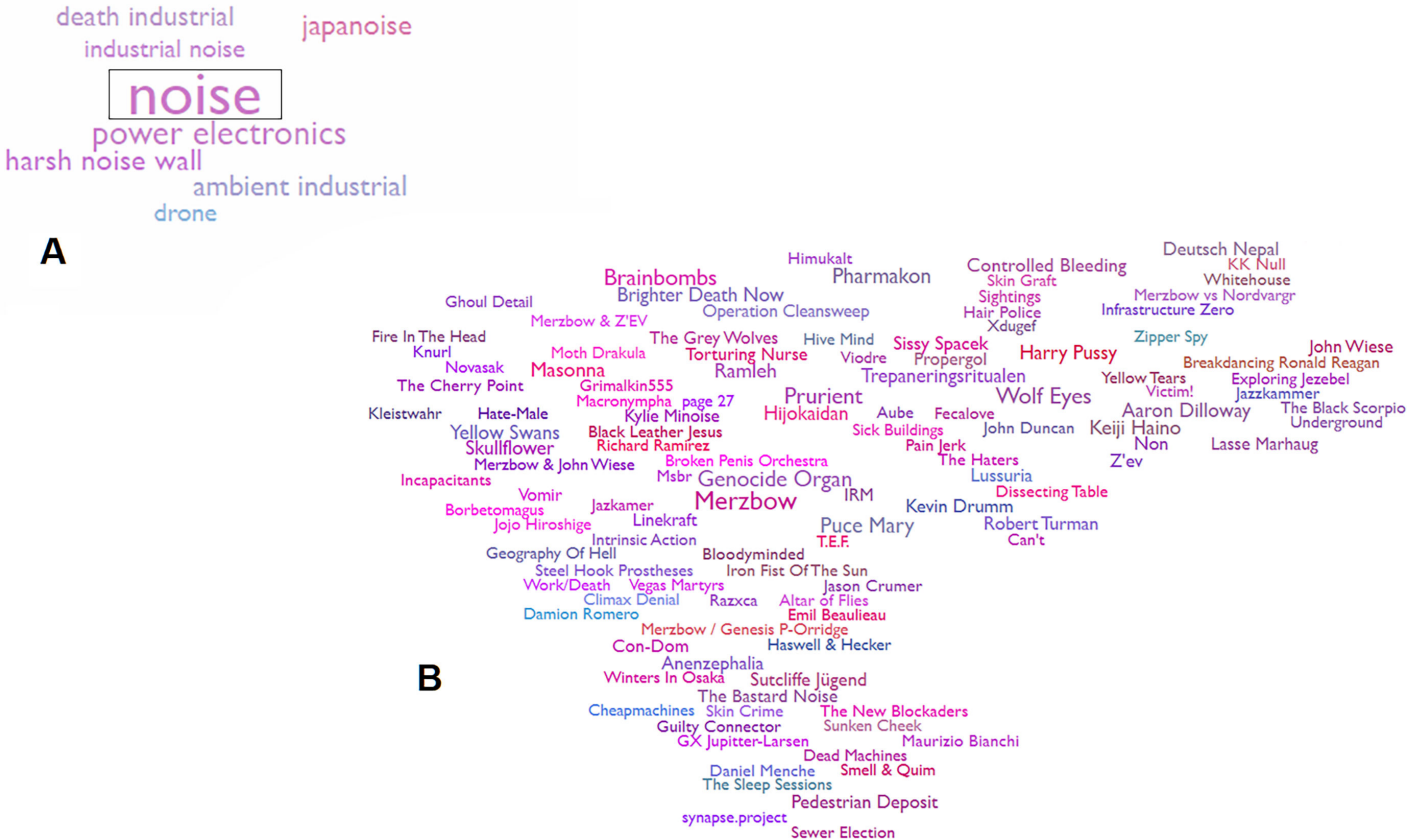
Algorithmically generated graph of music genre space and associated artists: (A) music genre *noise* surrounded by related subgenres genres, (B) music artists associated with *noise* genre. Drawn from the website ‘Every Noise at Once’ (https://everynoise.com/)

Cultural musical evolution is accelerating, which is changing the categories of phenomena typically considered ‘musical’ as well as the methods we use to generate that music. Technological advances not only change the ways music is made but also the ways that social groups of musicians interact. With profound increases in the size of musical cultural groups displaced in time and physical proximity, the means for transmitting trait values also increase dramatically. We now have musical creators who have abandoned the common dimensions of rhythm and melody in favour of pure spectral manipulation. Since the advent of distortion in rock, its use has increased both in the musical contexts that its used in (i.e. to all instruments and singing) and the extent to which heavy saturation is applied (i.e. reducing periodicity and adding inharmonic overtones). Figure 1B provides an example of a 6 min noise composition by the artist *Merzbow* that does not contain any discernible rhythmic or melodic structure. Rather, the piece incorporates manipulations of distortion with occasional rapid spectral sweeps and other noisy elements. A variety of artists produce music of this sort, using noise to construct long pieces that can also include subtle rhythmic and melodic components. Figure 4 shows an adjusted scatterplot map of artists in the ‘noise’ genre, generated through an algorithm based on genre distinctions drawn from Spotify. Even within groupings of musical pieces lacking rhythm and melody, users distinguish between spectral variants that many casual listeners would be unable to parse. The transmission dynamics described in our model can lead to such a scenario given enough time and the right conditions. These same dynamics, albeit with much more complex factors, likely underlie all musical innovation and evolution.

## Data Availability

Code for model presented in the electronic supplementary material. Supplementary material is available online [105].
